# Early host–parasite interaction models reveal a key role for fibrinolysis in *Fasciola hepatica* intestinal migration

**DOI:** 10.1186/s13071-025-06992-9

**Published:** 2025-09-24

**Authors:** Judit Serrat, Marta López-García, María Torres-Valle, Verónica Molina-Hernández, María Teresa Ruiz-Campillo, Mar Siles-Lucas, Javier González-Miguel

**Affiliations:** 1 Laboratory of Helminth Parasites of Zoonotic Importance (ATENEA), Institute of Natural Resources and Agrobiology of Salamanca (IRNASA-CSIC), Salamanca, Spain; 2 Departamento de Anatomía y Anatomía Patológica Comparadas y Toxicología, UIC Zoonosis y Enfermedades Emergentes ENZOEM, Facultad de Veterinaria, Universidad de Córdoba, Córdoba, Spain; 3 Departamento de Sanidad Animal (Parasitología), UIC Zoonosis y Enfermedades, Emergentes ENZOEM, Facultad de Veterinaria, Universidad de Córdoba, Córdoba, Spain

**Keywords:** Fasciola hepatica, Fibrinolysis, Plasminogen, Plasmin, Migration

## Abstract

**Background:**

*Fasciola hepatica* is the most common etiologic agent of fasciolosis, a parasitic disease that affects millions of ruminants worldwide and a zoonotic human infection of public health concern. Upon ingestion of infective metacercariae, *F. hepatica* newly excysted juveniles (FhNEJ) emerge in the duodenum and cross the intestinal wall to initiate a migration route that culminates with their establishment within the hepatic bile ducts. The ability of FhNEJ to exploit the broad-spectrum activities of host plasmin, the central protease of the fibrinolytic system, has been proposed as a strategy employed by these parasites to migrate across the intestinal wall while minimising energy expenditure.

**Methods:**

Mouse intestinal epithelial cells (mPSIEC) were stimulated with FhNEJ and plasminogen (PLG), the zymogen of plasmin, to understand whether FhNEJ-stimulated plasmin generation modulates processes relevant to parasite migration through the intestinal wall, including extracellular matrix (ECM) degradation and the secretion of ECM-degrading enzymes. Plasmin-mediated cellular responses were further examined by proteomic analysis of mPSIEC whole-cell lysates. In parallel, the contribution of the fibrinolytic system in FhNEJ migration was studied in vivo by infecting mice with *F. hepatica* metacercariae following pharmacological inhibition of fibrinolysis.

**Results:**

Co-stimulation of mPSIEC with FhNEJ and PLG led to increased plasmin generation in the intestinal pericellular space, which was associated with enhanced collagen degradation and secretion of the urokinase-type plasminogen activator. In addition, using independent cell culture replicates and a stringent statistical pipeline, we identified a robust set of differentially expressed proteins in mPSIEC following stimulation with FhNEJ and PLG. These proteins were involved in cell adhesion, migration, ECM remodelling, immune evasion and fibrinolysis. Despite inter-experimental variability, FhNEJ migration in mice was reduced upon pharmacological inhibition of fibrinolysis, supporting the contribution of host fibrinolysis to parasite invasion in vivo.

**Conclusions:**

Altogether, this work provides unprecedented insights into the role of the host fibrinolytic system to FhNEJ migration across mammalian host tissues, thereby advancing our understanding of host–parasite relationships during early stage fasciolosis and highlighting interesting directions for future research in this area.

**Graphical Abstract:**

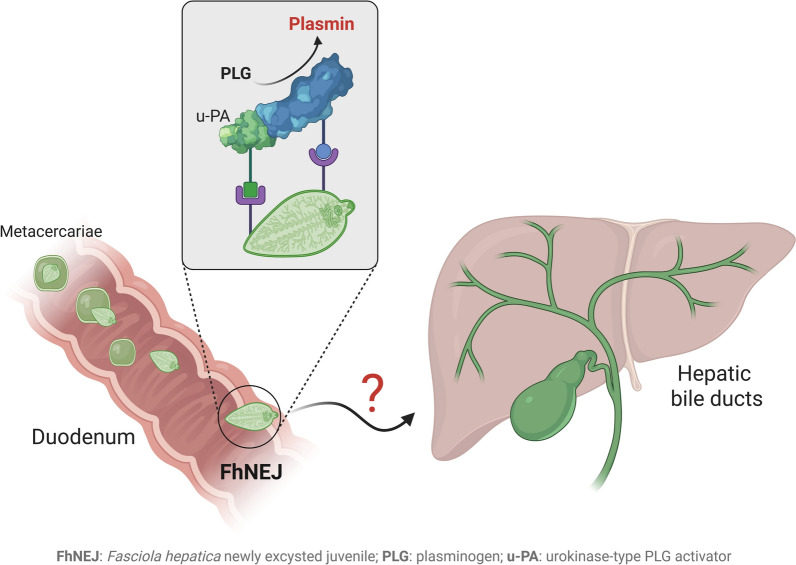

**Supplementary Information:**

The online version contains supplementary material available at 10.1186/s13071-025-06992-9.

## Background

A paradigmatic example of a host–parasite relationship is the ability of parasites to engage with the fibrinolytic system of their definitive hosts, a strategy that is considered a key aspect of parasite dissemination and survival within the mammalian organism [1–3]. The most common mechanism of interaction between parasites and host fibrinolysis is through the expression of lysine-rich proteins at the host–parasite interface – either on the parasite surface or secreted into the tissue microenvironment surrounding the parasite – that interact with plasminogen (PLG), the main zymogen of the fibrinolytic pathway. Upon binding to its receptors (PLG-Rs), PLG undergoes a conformational change that exposes the target sites of its activators, the proteases tissue-type and urokinase-type PLG activators (t-PA and u-PA, respectively), which convert PLG into its catalytically active form, the serine protease plasmin [4].

The classical function of the fibrinolytic system is to degrade the fibrin network that stabilises blood clots in the vascular endothelium [4]; however, owing to the broad spectrum of plasmin substrates, this system also participates in multiple biological processes that are both unrelated and independent of fibrin degradation and blood clot removal, including cell migration [5, 6]. Plasmin facilitates cell migration by degrading different components of the extracellular matrix (ECM), including collagen, laminin, fibronectin and proteoglycans [7–11], as well as by cleaving and activating the zymogens of different metalloproteinases (MMPs), which are considered key regulators of ECM remodelling during homeostasis, development and disease [12]. In addition, fibrinolysis-unrelated substrates of plasmin also include complement molecules and immunoglobulins [13]. On the basis of this, the stimulation of plasmin generation from host PLG within the tissue microenvironment surrounding parasites has been postulated as a mechanism facilitating immune evasion and their migration through host tissues [2, 3, 13].

*Fasciola hepatica* is one of many parasites whose interaction with the host fibrinolytic system has been experimentally shown [14–18]. *F. hepatica* is a helminth trematode with an indirect life cycle that includes freshwater snails as intermediate hosts, where the asexual phases of the parasites develop, and a mammalian definitive host, where adult flukes mature. Definitive hosts, typically ruminants and humans, become infected by ingesting aquatic plants contaminated with infective metacercariae, which excyst in the duodenum and release the newly excysted juveniles (FhNEJ). Shortly after excystment, FhNEJ cross the intestinal wall, and the juvenile flukes subsequently migrate through the peritoneum and liver parenchyma before entering the major hepatic bile ducts, where they mature into adults and produce fertilised eggs that are shed with the faeces [19]. *F. hepatica* is the most widespread etiologic agent of fasciolosis, a chronic and debilitating disease that affects millions of ruminants worldwide, thereby representing an economically important condition of domestic livestock that poses major threats to animal welfare and global food security [20, 21]. Since cases of human infection are predominantly concentrated in tropical areas of the globe, human fasciolosis is categorised by the Word Health Organization as a food-borne neglected tropical disease of growing public health concern requiring targeted intervention for its elimination [22, 23].

Using in vitro assays, we and others have previously shown that FhNEJ interact with the host fibrinolytic system by multiple mechanisms. These include the expression of proteins that bind to PLG, the precursor zymogen of u-PA (pro-u-PA), or both at the host–parasite interface [14–16, 18], as well as the expression of proteases that can stimulate pro-u-PA conversion into its catalytically active form, which further potentiates plasmin generation from host PLG [15]. Notably, FhNEJ-stimulated plasmin generation contributes to the endogenous capacity of these parasites to degrade laminin [24], one of the major components of the intestinal basement membrane [25], suggesting that the interaction between FhNEJ and host fibrinolysis may facilitate their migration across the intestinal wall. Since FhNEJ trans-intestinal migration is considered a crucial step for infection success, a deeper understanding of the mechanisms governing this process, including the contribution of the host fibrinolytic system, may offer potential avenues for developing novel therapeutic and control strategies against this widespread parasite [26].

Although the interaction between parasites and the host fibrinolytic system is well-established [1–3], only a minority of studies have performed functional assays using physiologically-relevant experimental models to assess whether it serves any physiological roles [1]. The abovementioned interplay between *F. hepatica* and the host fibrinolytic system exemplifies this gap. In the present study, we addressed this limitation by exploring the functional relevance of the interaction between FhNEJ and host fibrinolysis using a co-culture system of epithelial cells derived from the mouse small intestine, which simulates the first contact between FhNEJ and host tissues [27, 28], and a mouse model of early stage fasciolosis [29].

## Methods

### Culture of mouse primary small intestinal epithelial cells

C57BL/6 mouse primary small intestinal epithelial cells (mPSIEC; Cell Biologics) were cultured as previously described [27, 28] and in accordance with the manufacturer’s instructions. Briefly, cells were plated in 6 cm^2^ dishes (Corning) pre-coated with a gelatin-based coating solution (Cell Biologics) and grown in complete epithelial cell medium (Cell Biologics) in a humidified, 5% CO_2_ atmosphere at 37 ℃. Cell medium was replaced every 48 h, and cells were trypsinised and split at a 1:3 ratio when confluence was reached following standard cell culture procedures.

### Excystment of *F. hepatica* metacercariae

*F. hepatica* metacercariae (Italian strain, Ridgeway Research Ltd) were excysted as previously described [14]. Briefly, metacercariae were incubated for 1 h at 37 °C in a solution containing CO_2_ and 0.02 M sodium dithionite (Sigma), followed by three washes with distilled water and incubation in excystment medium [Hank’s balanced salt solution (Sigma) supplemented with 10% lamb bile (obtained from a local abattoir) and 30 mM HEPES (Sigma) at pH 7.4] at 37 °C. FhNEJ were manually recovered under a stereomicroscope using a 20 μL pipette every hour after addition of excystment medium, immediately transferred to a clean plate containing complete epithelial cell medium, and incubated in a humidified, 5% CO_2_ atmosphere at 37 °C to induce recovery for 1 h prior to transfer to mPSIEC cultures.

### In vitro interaction model

FhNEJ were co-cultured with mPSIEC as previously described [27, 28], with some modifications. At passage 5, mPSIEC were cultured in 6-well plates until confluence was reached. At that point, 200 FhNEJ were added to the corresponding wells in 2 mL of complete epithelial cell medium in the presence or absence of 10 µg/mL of human PLG (Origene). A condition where cells were co-incubated with 200 FhNEJ, PLG (10 µg/mL), and 50 mM of the lysine analogue 6-aminocaproic acid (ε-ACA; Sigma) was included to assess whether the mechanism of FhNEJ-induced plasmin generation is lysine-dependent. Cells left unstimulated, cells stimulated only with 200 FhNEJ, and cells stimulated only with PLG (10 µg/mL) were used as controls. The parasites were separated from the cells 24 h after stimulation, and whole-cell lysates and cell culture supernatants were harvested for downstream analyses. First, 1 mL of each culture supernatant was carefully aspirated and transferred to 1.5 mL tubes, followed by centrifugation for 5 min at 13,000 ×*g* and 4 °C. The pellets containing cell debris were discarded, and clean supernatants were frozen at −80 °C until use. Whole-cell lysates were obtained by washing the cells in pre-warmed phosphate-buffered saline (PBS) followed by scraping in 200 µL of Radioimmunoprecipitation assay (RIPA) buffer (Sigma). Whole-cell lysates were transferred to 1.5 mL tubes, vigorously vortexed for 30 s and centrifuged for 5 min at 13,000 ×*g* and 4 °C. Pellets containing the cell debris were discarded, and supernatants were transferred to clean 1.5 mL tubes and stored at −80 °C until use. Protein concentrations were determined using the Pierce BCA Protein Assay kit (Thermo Fisher) and ranged between 1.03 and 1.37 mg/mL protein, depending on the sample. Each experimental condition was conducted in triplicate.

### Plasmin activity in cell culture supernatants

Plasmin activity in cell culture supernatants collected from mPSIEC in the in vitro interaction model was assayed by measuring the amidolytic activity of generated plasmin on a plasmin-specific chromogenic substrate, as previously described [14], with minor modifications. In every well of a transparent, flat-bottom, 96-well microtiter plate, equal volumes of samples were mixed with 1 mM of d-Val-Leu-Lys 4-nitroanilide dihydrochloride chromogenic substrate (S-2251, Sigma) in a total volume of 100 µL of PBS. The microplates were incubated for up to 24 h at 37 °C, and substrate cleavage was assessed by measuring absorbance at 405 nm every hour in a Multiskan GO spectrophotometer (Thermo Fisher). Wells containing 0.02 µM of plasmin (Origene) instead of cell culture supernatant were used to control for substrate specificity. All the reactions were performed in technical triplicate.

### Analysis of u-PA levels in cell culture supernatants

The levels of u-PA in cell culture supernatants (100 µL) were measured by enzyme-linked immunosorbent assay (ELISA) using the Mouse PLAU/Upa (Urokinase-Type Plasminogen Activator) ELISA kit (FineTest) and following the manufacturer’s instructions. The reaction was stopped 11 min after addition of the 3,3′,5,5′-tetramethylbenzidine substrate, and the amount of u-PA in the samples was quantified by fitting a regression line to the linear portion of a standard curve with known u-PA concentrations. All the reactions were performed in technical duplicate.

### Analysis of total collagen in cell culture supernatants

The levels of total collagen in cell culture supernatants (20 µL) were measured by enzymatic digestion of collagen into *N*-Gly terminal peptides that react with a dye reagent to form a fluorescent complex, using the Collagen Assay Kit (Assay Genie) and following the manufacturer’s instructions. The amount of collagen in the samples was quantified by fitting a regression line to the linear portion of a standard curve with known collagen concentrations. All the reactions were performed in technical duplicate.

### Zymography

The ability of plasmin to degrade the mPSIEC coating solution was measured by preparing 10% sodium dodecyl sulfate (SDS)-polyacrylamide gels using standard protocols but replacing water with the mPSIEC coating solution (Cell Biologics). Next, different amounts of plasmin (Origene) were mixed with non-reducing sample buffer (125 mM Tris–HCl, 4% SDS, 20% glycerol, 0.01% bromophenol blue) in a total volume of 18 µL, and samples were loaded onto the gel in duplicate. Electrophoresis was performed at a constant current of 15 mA for 20 min followed by 30 mA. After electrophoresis, the gel was soaked twice in wash buffer (5 mM CaCl_2_, 2% Triton-X, pH 7.4) for 30 min at room temperature, followed by overnight incubation at 37 °C in digestion buffer (5 mM CaCl_2_, 0.1 M glycine, pH 8.4) [30]. All the incubations were performed with mild shaking. After incubation, the gels were stained with Coomassie brilliant blue staining, following standard procedures, and imaged in a Chemidoc MP Imaging System (BioRad).

### Analysis of MMP levels in cell culture supernatants

The levels of MMP-2, MMP-3, MMP-8, pro-MMP-9 and MMP-12 in mPSIEC culture supernatants (25 µL) were analysed on a Luminex xMAP platform using a 5-plex mouse MMP magnetic bead panel (Millipore) and in accordance with the manufacturer’s instructions. Multiplex assays were analysed on a Liquichip Luminex 100 Liquid Array Multiplexing System XYP Lab (Luminex Corporation, Qiagen) with Luminex Xponent 3.1 software (Luminex Corporation), following the manufacturer’s technical guidelines. Data analysis was performed with the Milliplex Analyst 5.1 Software (Merck). All the reactions were performed in technical duplicate. The experiment and analysis were conducted at the Flow Cytometry Facility of the National Centre for Biotechnology (CNB-CSIC, Madrid, Spain).

### Proteomic analysis of mPSIEC whole-cell lysates

#### Sample preparation

Protein concentrations were determined using the Macherey–Nagel Protein Quantification Assay (Macherey–Nagel), and equal amounts of protein were reduced with 2 mM dithiothreitol at 60 °C for 20 min, and alkylated with 5 mM iodoacetamide diluted in 50 mM ammonium bicarbonate at room temperature for 30 min. Next, proteins were purified using the SP3 protocol [31, 32] to eliminate detergents prior to in-solution protein digestion. Purified proteins were digested overnight at 37 °C with 100 ng of sequencing-grade trypsin (Promega) diluted in 50 mM ammonium bicarbonate, and the reaction was stopped by addition of 0.1% trifluoroacetic acid.

#### Liquid chromatography (LC)–MS/MS analysis

Liquid chromatography (LC) was performed by loading 200 ng of digested peptides (diluted in 20 µL of 0.1% formic acid) onto an Evotip Pure tip (EvoSep) following the manufacturer’s instructions, followed by separation using the Evosep One system on an analytical Performance 15 cm × 150 μm column with a particle size of 1.5 μm (Evosep). The eluted peptides were ionised in a CaptiveSpray with 1700 V at 200 °C, and tandem mass spectrometry analysis (MS/MS) was performed with a TimsTOF fleX mass spectrometer (Bruker) in data-independent acquisition Parallel Accumulation-Serial Fragmentation (diaPASEF) mode.

#### Data processing and protein quantification

The PASER system (Bruker) was used to send the raw data for subsequent analysis and quantification with DIA-NN version 1.8 software (https://github.com/vdemichev/DiaNN). First, an in silico-predicted spectral library was built from the Uniprot *Mus musculus* database using DIA-NN version 1.8, using QuantUMS (high precision) as the quantification strategy. After raw data normalisation and quantification, proteins were filtered at < 1% false discovery rate (FDR) to ensure confidence in protein identification. The proteomic analysis was carried out at the Proteomics Unit of the Central Support Service for Experimental Research (SCSIE) of the University of Valencia (Spain), which is part of the Instituto de Salud Carlos III (ISCIII) ProteoRed Proteomics Platform.

#### Statistical analysis

Peak intensities of all the conditions were transformed to the logarithm with base *e* using the log1p function in R. Next, a stringent statistical pipeline employing three distinct methodologies was applied to identify differentially expressed proteins (DEPs) with high robustness, as previously described [33]. First, the glmnet package in R was employed to fit an elastic-net regularised regression model to select those variables (proteins) that better explained each experimental condition [34] (Supplementary Fig. S1). To refine the model, the nearZeroVar function from the caret package in R was applied to identify variables with near-zero variance, indicating very low variability across observations. These proteins were discarded from subsequent analysis. The values of the regularisation parameters (alpha and lambda) were optimised with the train function of the caret package through cross-validation re-sampling to obtain the regularised model that best fitted our data. The log-transformed expression values of the selected proteins were standardised through *Z*-score normalisation to ensure direct comparability of protein expression levels across samples, followed by representation in heatmaps. Second, feature selection by elastic-net was validated through partial least squares discriminant analysis (PLS-DA) using the mixOmics package in R [35] (Supplementary Fig. S2). This package contains the ‘variable importance in the projection’ (vip) function, which estimates the importance of each variable in the projection. When vip > 1.5, the influence of the variable on the response is very high [33]. Third, differential expression analysis between selected pairs of experimental conditions was performed using the limma package in R to get fold-change (FC) differences in protein expression along with the corresponding *P*-values [36]. Only proteins that were selected by both elastic-net and PLS-DA (vip > 1.5) and exhibited a significant fold-change (*P*-value < 0.05), as calculated by limma, were considered differentially expressed. This analysis was performed by the Statistics and Omics Data Analysis Unit of the Central Support Service for Experimental Research (SCSIE) of the University of Valencia, Spain.

#### Analysis of interaction effects

To identify proteins whose differential expression was specifically driven by the interaction of FhNEJ and PLG, rather than by either factor alone, an interaction effect analysis was conducted on the list of DEPs identified using the abovementioned statistical pipeline in the untreated versus FhNEJ + PLG comparison. To ensure that only proteins influenced by the interaction between FhNEJ and PLG were retained, proteins that were also differentially expressed in either the untreated versus FhNEJ or untreated versus PLG comparisons were excluded unless their expression in the combined treatment deviated significantly from the expected additive effect. This approach is conceptually similar to established methodologies in gene interaction studies aimed at detecting synergistic genetic perturbations, where interaction effects are assessed by comparing the observed combined effects to the expected additive effects of individual perturbations [37, 38]. To this end, an expected additive FC was computed as the sum of the individual effects of FhNEJ and PLG, on the basis of FC values obtained from the untreated versus FhNEJ and untreated versus PLG comparisons. An interaction score was then calculated as the difference between the observed FC in the untreated versus FhNEJ + PLG comparison and the expected additive FC. Proteins common to all three comparisons were considered to be differentially expressed as a result of the interaction between FhNEJ and PLG only if their absolute interaction score exceeded a threshold. To avoid arbitrary thresholding, interaction scores were filtered using the 1.5 × IQR rule, where IQR is the interquartile range, a standard method for outlier detection based on Tukey’s definition of extreme values [39]. Therefore, the interaction threshold was defined as: median interaction score ± (1.5 × IQR); where the median interaction score is the median of all the interaction scores calculated across proteins in the untreated versus FhNEJ + PLG condition. This analysis ensured that only proteins exhibiting substantial interaction effects – whether synergistic or antagonistic – were retained for further investigation. In addition to this analysis, a complementary approach was performed to identify proteins whose differential expression in the FhNEJ + PLG condition, when compared with FhNEJ alone, could not be explained solely by the effect of PLG. In this case, proteins were filtered on the basis of their FC values in the untreated versus PLG comparison. A threshold was defined as the median absolute FC ± (1.5 × IQR) in this comparison, capturing the typical range of PLG-induced expression changes. Only proteins in the FhNEJ versus FhNEJ + PLG comparison with an absolute FC exceeding this threshold were retained.

### In vivo model

A total of 20 wild-type, 49–55-day-old female C57BL/6 mice (Charles River Laboratories) were divided in two groups: one group (*n* = 10) was injected intraperitoneally with mouse PLG activator inhibitor-1 (PAI-1) recombinant stable mutant (50 µg per mouse; Innovative Research) to inhibit the fibrinolytic route, as previously described [40]; and the control group (*n* = 10) was injected intraperitoneally with sterile phosphate-buffered saline (PBS). All mice were challenged with *F. hepatica* metacercariae 30 min after injection, following a protocol recently described by our lab [29]. First, mice were orally infected with 125 *F. hepatica* metacercariae as follows: metacercariae were resuspended in a total volume of 50 µL of PBS, and oral infection was performed using a sterile 20–200 µL pipette tip that was trimmed to 0.5 cm and pre-washed with PBS containing 0.01% Triton X-100 to prevent the metacercariae from sticking to the plastic tip. Second, 8 days after challenge with *F. hepatica* metacercariae, mice were euthanised after 24 h of fasting through CO_2_ overdose followed by cervical dislocation. Prior to dissection, 2 mL of sterile PBS were injected into the peritoneal cavity, taking care to avoid puncturing any organs. Peritoneal fluid was then collected by making a small abdominal incision, followed by careful aspiration with a pipette tip. The liquid was transferred to 1.5 mL tubes and immediately frozen at −80 °C. For the recovery of juvenile worms from the hepatic parenchyma, livers were carefully dissected with tweezers under a stereomicroscope, and the number of juvenile parasites was manually counted by independent, trained, and experimental group-blinded researchers that were unfamiliar with the objective of the experiment. The experiment was conducted over 2 consecutive days, using five mice per group per day, with the same reagents and personnel. Animal handling was performed by qualified staff at the Experimental Animal Facility of the University of Córdoba (Spain). Mice were housed in groups of four per cage, with aspen wood bedding, and provided food and water ad libitum on a standard dry pellet diet for rodents. The experiment was conducted after 1 week of acclimatisation at 22 ± 3 °C, 50–60% relative humidity, and a 12 h light/dark cycle.

### PAI-1 and t-PA activity assays

The activities of PAI-1 and t-PA in peritoneal fluids of mice were measured using the commercially available Mouse active PAI1 ELISA Kit (Innovative Research) and Mouse Active tPA ELISA Kit (Innovative Research), following the manufacturer’s instructions. The amounts of active PAI-1 and t-PA in the samples were quantified by fitting a regression line to the linear portion of a standard curve with known active PAI-1/t-PA concentrations. All the reactions were performed in technical duplicate.

### Statistical analysis

Plots were created, and statistical analyses were performed using Prism 10 software (GraphPad Software). Unless otherwise stated, graph bars represent the mean of three cell culture replicates, each calculated as the average of two or three technical replicates. Error bars represent either the standard error of the mean (SEM) or the standard deviation (SD) to reflect the precision of the sample mean as an estimate of the population mean or the variability among individual technical measurements, respectively. The data from the in vivo experiment were analysed using a two-way analysis of variance (ANOVA), considering both treatment groups and the day on which the experiment was conducted as factors. On the basis of the results of this analysis, the data was plotted separately for each day and presented as mean ± SEM. Since normality in data distribution cannot be confidently determined with small sample sizes [41], non-parametric methods were chosen to determine statistically significant differences between experimental conditions across biological replicates. Specifically, the Mann–Whitney *U* test was used to assess differences between two experimental conditions; and comparisons between more than two experimental conditions were performed via Kruskal–Wallis test followed by Dunn’s post hoc analysis of pairwise comparisons. Unless otherwise stated, the threshold for significance was set at *α* ≤ 0.1.

## Results

### FhNEJ stimulate plasmin generation in the pericellular space of mouse intestinal epithelial cells

**Fig. 1:**
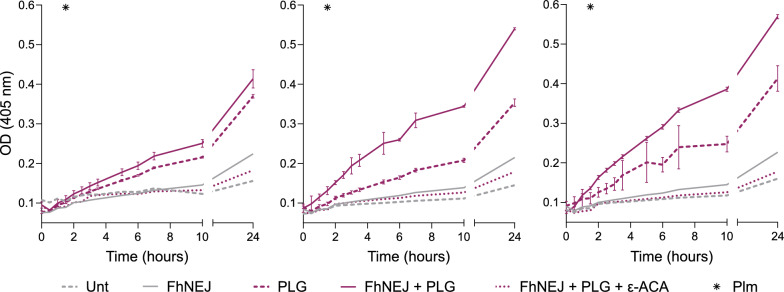
FhNEJ stimulate plasmin generation from host PLG in the pericellular space of mPSIEC. mPSIEC cultures were incubated with FhNEJ and PLG and plasmin generation on cell culture supernatants (optical density (OD) at 405 nm) was assessed 24 h after stimulation in an enzymatic assay by mixing equal amounts of cell culture supernatants with a plasmin-specific chromogenic substrate. Cells left untreated (Unt) or those that were single-treated with either FhNEJ or PLG served as negative controls for FhNEJ-induced plasmin generation, and cells incubated with FhNEJ and PLG in the presence of ε-ACA were used to assess whether FhNEJ-induced plasmin generation was mediated by lysine residues. A condition was included in where the plasmin-specific chromogenic substrate was incubated with recombinant plasmin (Plm) instead of cell culture supernatant to control for substrate specificity. The results from three independent cell culture replicates are shown. Lines indicate the mean of three technical replicates ± SD. Error is displayed only for the PLG-only and FhNEJ + PLG conditions for simplification purposes

To study whether FhNEJ stimulate plasmin generation from host PLG in an in vitro system of host–parasite interactions [27, 28], mPSIEC were co-incubated for 24 h with FhNEJ in the presence of host PLG and without the exogenous addition of PLG activators. A condition where cells were co-incubated with FhNEJ, PLG, and the lysine analogue ε-ACA was included to assess whether FhNEJ-induced plasmin generation was mediated by lysine residues present in FhNEJ-derived PLG-interacting proteins, as previously described [14]. Plasmin generation in cell culture supernatants of mPSIEC was assessed using a plasmin-specific chromogenic substrate, which revealed that cells incubated with PLG alone showed a small degree of plasmin generation that was increased in the presence of FhNEJ. This effect was observed across three independent cell culture replicates and was completely abrogated in the presence of ε-ACA, indicating that the observed increased plasmin generation was mediated by lysine residues (Fig. 1).

### FhNEJ increase u-PA levels in mPSIEC culture supernatants and promote collagen degradation in a PLG-dependent manner

**Fig. 2:**
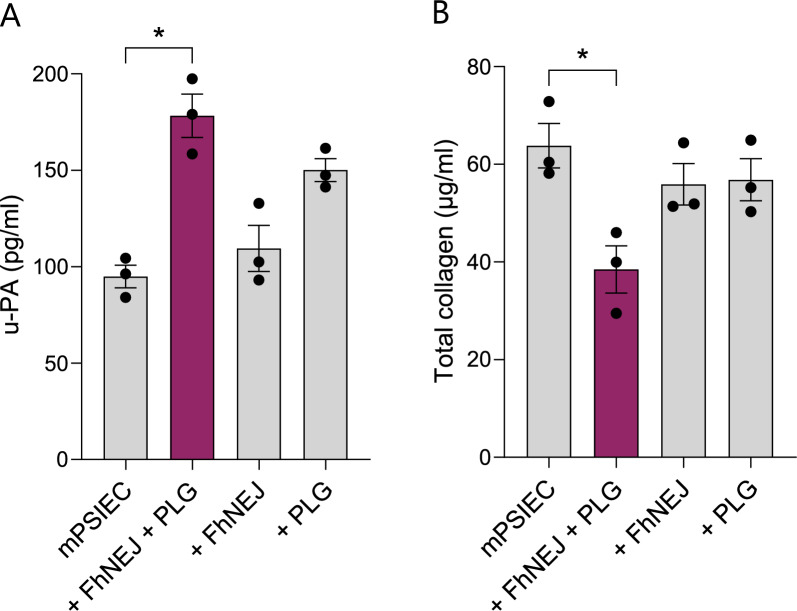
FhNEJ stimulate u-PA release and collagen degradation by mPSIEC in the presence of PLG. mPSIEC cultures were stimulated with FhNEJ and PLG, and the levels of u-PA (**A**) and total collagen (**B**) were analysed in cell culture supernatants 24 h after stimulation by ELISA (**A**) or by measuring the amount of *N*-Gly terminal peptides generated after enzymatic digestion with a collagenolytic enzyme (**B**). In both panels, cells left untreated or single-treated with either FhNEJ or PLG served as negative controls. Data points represent the value obtained for each independent cell culture replicate, calculated as the average of two technical replicates, and bars represent the mean ± SEM. Only statistically significant differences are shown (^*^P ≤ 0.1; Kruskal–Wallis test followed by Dunn’s post hoc test for pairwise comparisons)

We next addressed how mPSIEC respond to the presence of FhNEJ in terms of u-PA release and collagen degradation, and whether these responses would be modulated by the interaction between FhNEJ and PLG. The levels of u-PA in cell culture supernatants of mPSIEC left unstimulated, stimulated with FhNEJ and PLG, or stimulated with either component alone were assessed through ELISA, and total collagen in these samples was quantified by measuring the amount of *N*-Gly terminal peptides generated upon treatment of the samples with a collagenolytic enzyme. These experiments revealed that, in contrast to cell culture supernatants from mPSIEC treated individually with either FhNEJ or PLG, cell culture supernatants from mPSIEC stimulated with both FhNEJ and PLG exhibited a statistically significant increase in u-PA levels (Fig. 2A) and a decrease in collagen (Fig. 2B; Supplementary Fig. S3A) compared with supernatants from untreated mPSIEC. In line with this, standard zymography performed with increasing amounts of recombinant plasmin revealed that this protease degrades the ECM coating solution used to culture mPSIEC (Supplementary Fig. S3B). Altogether, these results showed that the stimulation of mPSIEC with both FhNEJ and PLG results in increased u-PA release and ECM degradation in this in vitro cell system.

### Regulation of MMP levels in mPSIEC culture supernatants by FhNEJ

**Fig. 3:**
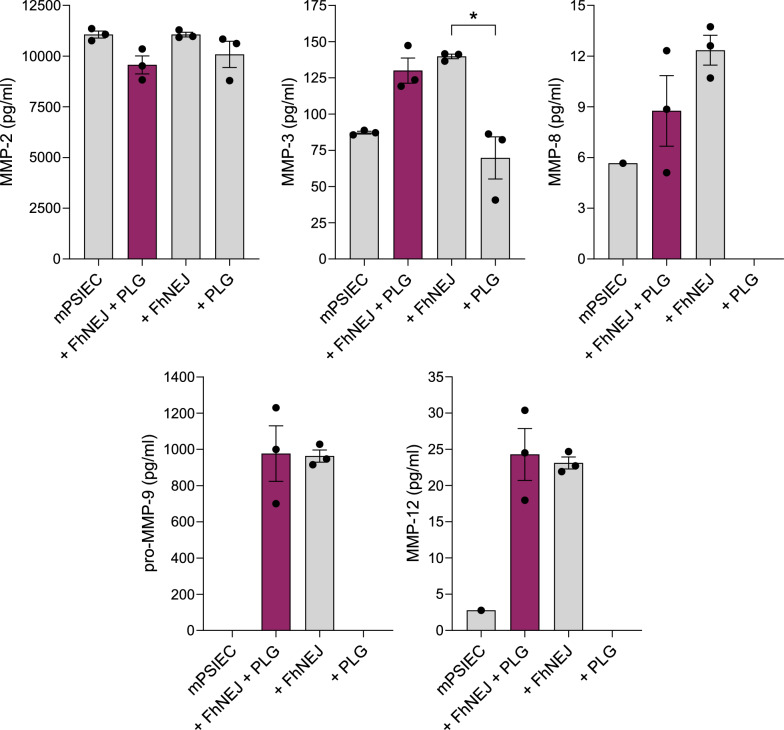
Increased secretion of MMP-3, MMP-8, MMP-12 and pro-MMP-9 by mPSIEC upon stimulation with FhNEJ. mPSIEC cultures were stimulated with FhNEJ and PLG, with either component alone, or left unstimulated, and the levels of MMP-2, MMP-3, MMP-8, MMP-12 and pro-MMP-9 were simultaneously assayed using multiplexed assays. In all graphs, data points represent the value obtained for each independent cell culture replicate, calculated as the average of two technical replicates, and bars represent the mean ± SEM. Only statistically significant differences are shown (^*^P ≤ 0.1; Kruskal–Wallis test followed by Dunn’s post hoc test for pairwise comparisons). Note that statistical analysis could not be performed for MMP-8, MMP-12 and pro-MMP-9 owing to some MMP levels being below the detection limit of the technique (PLG-only in MMP-8 analysis; PLG-only in MMP-12 analysis; and untreated and PLG-only in pro-MMP-9 analysis)

We went on to evaluate whether FhNEJ influence the secretion of different ECM-degrading MMPs by mPSIEC, and whether this would be influenced by PLG. To that end, the levels of MMP-2, MMP-3, MMP-8, MMP-12, and pro-MMP-9 were analysed through multiplexed assays in cell culture supernatants of mPSIEC left unstimulated, stimulated with FhNEJ and PLG, or stimulated with either component alone (Fig. 3). This experiment revealed that MMP-2 and pro-MMP-9 are the MMPs showing the most abundant concentrations in the cell culture supernatants of mPSIEC. Specifically, the levels of MMP-2 were similar across the different experimental conditions, whereas those of pro-MMP-9 were only detectable in the conditions including FhNEJ (FhNEJ-only and FhNEJ + PLG). Similar results were obtained for MMP-3, MMP-8 and MMP-12, despite their levels in mPSIEC culture supernatants being much lower than those observed for MMP-2 and pro-MMP-9. Overall, although some experimental conditions could not be quantified owing to values falling below the detection limits of the technique, these results indicate that MMP levels in cell culture supernatants substantially increase upon incubation of mPSIEC with FhNEJ, regardless of PLG availability.

### Proteomic profiling reveals mPSIEC responses driven by the interaction between FhNEJ and host PLG

To characterise in greater detail how mPSIEC respond to FhNEJ and whether these responses are modulated by their interaction with host PLG, a proteomic analysis was conducted in whole-cell lysates from mPSIEC left unstimulated, stimulated with FhNEJ and PLG, or stimulated with either component alone. This analysis identified a total of 8136 proteins with FDR < 1% that were included in the differential expression analysis. We compared the protein expression profiles of untreated cells with cells stimulated with both FhNEJ and PLG to identify proteins that are differentially regulated by the interaction between both factors, which resulted in increased plasmin activity in the pericellular space (Fig. 1). To keep only proteins regulated by the combination of FhNEJ and PLG, and not by either component alone, DEPs identified in both the untreated versus FhNEJ and the untreated versus PLG comparisons that overlapped with DEPs identified in the untreated versus FhNEJ + PLG comparison were not considered as differentially regulated by FhNEJ + PLG unless their FC values showed an interaction effect. As a result of this interaction analysis, we identified a total of 84 DEPs in mPSIEC treated with FhNEJ and PLG that were specifically attributable to the combined effect of both components (Table 1).

**Table 1:** DEPs in untreated versus FhNEJ + PLG-treated mPSIEC

Genes	logFC	adjPVal	Genes	logFC	adjPVal
Cap1	−11.5641	1.16 × 10^−8^	Mrgbp	0.315304	0.041192
Eif1ad11	−10.8284	0.044884	Sgo2	0.321909	0.044884
Zfp606	−9.98762	1.16 × 10^−8^	Tmem184b	0.337123	0.042419
Serpinb5	−9.09	1.29 × 10^−5^	Cdca2	0.337796	0.044267
A130006I12Rik	−8.8194	6.9 × 10^−6^	Aurka	0.337847	0.037707
S1pr2	−8.79117	6.89 × 10^−5^	Glul	0.353169	0.029492
Pptc7	−7.5837	3.26 × 10^−7^	Blm	0.365228	0.032615
Tmem14c	−0.70435	0.044884	Actn4	0.36718	0.046035
Rab3b	−0.67455	0.031743	Rab38	0.367389	0.026216
Mmp11	−0.67003	0.046035	Aurkb	0.370126	0.047626
Dennd3	−0.4902	0.004343	Sash1	0.377697	0.044267
Desi2	−0.44875	0.044884	Rpl35	0.398069	0.012174
Dhx38	−0.44049	0.035042	Acsl4	0.398548	0.007476
Srsf2	−0.43185	0.041835	Cox6a1	0.405347	0.028322
Gorasp1	−0.38835	0.011505	Esco2	0.419492	0.018064
Ccn2	−0.37626	0.046035	0610010K14Rik	0.476796	0.044884
Atrx	−0.32326	0.04661	Arhgap20	0.48969	0.044267
Slc25a30	−0.31699	0.046035	Cmip	0.490543	0.046035
P4ha2	−0.26881	0.044267	Aven	0.490844	0.041192
Slc12a7	−0.2512	0.026216	Gmnn	0.495586	0.047626
Scel	−0.24909	0.044267	Cdc34	0.522914	0.040503
Gpam	−0.23204	0.046035	Suco	0.539981	0.018465
Sppl2a	0.191843	0.042419	Cetn2	0.553036	0.040283
Ska3	0.199186	0.0426	Nifk	0.619508	0.046035
Lama5	0.223652	0.040283	Fnip1	0.677284	0.006195
Slc9a3r2	0.224492	0.046404	Lrig1	0.745655	0.00283
Dvl2	0.239475	0.046035	Sema3a	0.831001	0.031743
Cdv3	0.244815	0.035681	Serpind1	0.858629	0.032615
Notch2	0.247477	0.022438	Fn1	0.888985	0.031603
Lamb1	0.250854	0.018889	Zfhx4	1.130508	0.010738
Prkd2	0.254831	0.048562	Thbs1	1.239887	0.035681
Diaph3	0.255139	0.018889	Serpinb7	1.278409	0.003469
Rnf4	0.25624	0.044267	A2m	1.423682	0.016865
Prr11	0.265523	0.04991	Itih4	1.425836	0.026216
Spdl1	0.271143	0.031743	Tmc5	1.469909	0.002405
Melk	0.27314	0.048562	C7	1.471351	0.003517
Calu	0.28254	0.022262	Dnah14	1.608509	0.026216
Fam50a	0.286497	0.011574	Mettl25	7.646163	4.28E-05
Mphosph10	0.292922	0.028712	Pigr	8.709757	4.38E-06
Msmo1	0.302965	0.031743	Sema3d	10.34484	3.39E-07
Ror1	0.311029	0.021179	Lamtor3-ps	12.05803	0.00283
Tns2	0.313294	0.017259	Alb	12.61229	2.51E-07

A negative fold-change indicates that proteins were overrepresented in FhNEJ + PLG-treated versus untreated mPSIEC. The opposite is true when the fold-change is positive

In addition to proteins presented in Table 1, we also considered as differentially regulated as a result of the interaction between FhNEJ and PLG those DEPs identified by comparing mPSIEC stimulated with FhNEJ + PLG with cells stimulated with only FhNEJ. Following a similar rationale as described above, we excluded proteins regulated by PLG alone by discarding those DEPs in the FhNEJ versus FhNEJ + PLG comparison that were common to those identified in the untreated versus PLG comparison unless their fold change under the FhNEJ + PLG condition exceeded the typical range of PLG-induced expression changes. By doing so, we identified a total of 15 proteins that were differentially regulated in mPSIEC as a result of the stimulation with both FhNEJ and PLG (Table 2). Notably, this analysis identified PLG (Plg) as significantly overrepresented in FhNEJ + PLG-treated cells compared with cells treated with FhNEJ alone, highlighting the accuracy of the approach. By combining both analyses, we identified 94 unique DEPs in double-treated mPSIEC, which were involved in cell adhesion and migration, ECM remodelling, mucosal immunity, and fibrinolysis. These included cyclase-associated protein 1 (Cap1), serpin b5, the Ras-related protein Rab3b, actinin-4 (Actn4), MMP-11 (Mmp11), the tissue inhibitor of metalloproteinase 3 (Timp3), fibronectin 1 (Fn1), semaphorin 3D (Sema3d), the polymeric immunoglobulin receptor (Pigr), the complement molecule C7, the sphingosine 1-phosphate receptor 2 (S1pr2), alpha-2 macroglobulin (A2m), and carboxipeptidase 2 (Cpb2), also known as the thrombin-activatable fibrinolysis inhibitor (TAFI).

**Table 2:** DEPs in FhNEJ-treated versus FhNEJ + PLG-treated mPSIEC

Genes	logFC	adjPVal
Cap1	−11.5928	2.45 × 10^−8^
Cpb2	−8.97008	9.76 × 10^−7^
Ube2l6	−8.30553	9.76 × 10^−7^
Qsox1	−9.00044	9.76 × 10^−7^
Sema3d	10.18779	9.76 × 10^−7^
Klhl30	10.2297	4.84 × 10^−6^
Chst2	7.69985	5.86 × 10^−6^
Timp3	−0.59491	0.004089
Rab3b	−0.50349	0.010792
Plg	−1.34697	0.014915
Dennd3	−0.42075	0.022473
Phip	−0.55656	0.025486
Ccn1	−0.45733	0.043756
Scel	−0.36049	0.043756
Slco2a1	−0.33878	0.044832

A negative fold-change indicates that proteins were overrepresented in FhNEJ + PLG-treated versus FhNEJ-treated mPSIEC. The opposite is true when the fold-change is positive

### The contribution of the fibrinolytic system to FhNEJ migration in a mouse model of fasciolosis

**Fig. 4:**
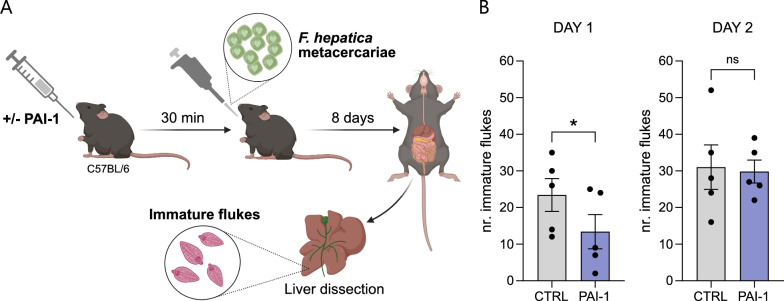
Study of the influence of the fibrinolytic system in FhNEJ migration in a mouse model of fasciolosis. (**A**) Schematic representation of the experimental design. Mice were injected with either PBS (control) or a recombinant, stable-mutant version of PAI-1 to inhibit the fibrinolytic route 30 min prior to infection with 125 *F. hepatica* metacercariae, as previously described [40]. Subsequently, 8 days after challenge with *F. hepatica* metacercariae, mice were euthanised, and the number of juvenile flukes in the livers were manually counted under a stereomicroscope. Created with Biorender.com. (**B**) Counts of juvenile flukes recovered from the livers of PAI-1-treated and control mice 8 days after challenge with *F. hepatica* metacercariae. Data points represent the number of juveniles recovered in the liver of each mouse, and bars indicate the mean ± SEM. Asterisks indicate significant differences between experimental conditions (ns, not significant; ^*^P ≤ 0.1; Mann–Whitney *U* test)

Finally, in an effort to understand the contribution of the host fibrinolytic system in the migration of *F. hepatica* juveniles during the earliest stages of infection in a physiologically relevant experimental system, we employed a mouse model of early stage fasciolosis [29] to quantify the amount of juvenile flukes that successfully migrate to the livers of control mice and mice whose fibrinolytic route had been pharmacologically inhibited prior to challenge with *F. hepatica* metacercariae (Fig. 4A). Owing to technical constraints, the experiment was performed across 2 consecutive days, employing five mice per group on each day. Statistical analysis using two-way ANOVA revealed that the day of the experiment resulted in statistically significant differences in FhNEJ counts (Supplementary Table S2), as PAI-1-treated mice from day 1 – but not control mice – exhibited significantly different amounts of liver-migrating flukes than their counterparts from day 2 (Supplementary Fig. S4). Therefore, PAI-1-treated mice could not be treated as true replicates, and data were subsequently plotted and analysed separately for each day. This analysis revealed that PAI-1 treatment prior to *F. hepatica* infection resulted in a significant decrease in the number of juvenile flukes recovered from the livers of infected mice 8 days after challenge with *F. hepatica* metacercariae, but only in the experiment conducted on day 1 (Fig. 4B). To assess potential differences in PAI-1 treatment efficacy across different experimental days, we measured the levels of fibrinolysis in the peritoneal fluids of all mice, which were collected immediately after euthanasia. However, no statistically significant differences were observed in either PAI-1 or t-PA activities between control and PAI-1-treated mice on any experimental day (Supplementary Fig. S5).

## Discussion

Aiming to understand the contribution of the fibrinolytic system in biological processes that might facilitate FhNEJ migration, commercially available primary intestinal epithelial cells derived from the mouse small intestine (mPSIEC) were stimulated with FhNEJ [27, 28] and PLG for 24 h, after which cell culture supernatants and mPSIEC whole-cell lysates were harvested for downstream analyses. In addition, mPSIEC that were left unstimulated or stimulated with either FhNEJ or PLG alone served as negative controls, and an additional subset of mPSIEC stimulated with FhNEJ, PLG, and the lysine analogue ε-ACA was used to assess whether plasmin generation depends on the presence of lysine residues in FhNEJ-derived PLG-interacting proteins, as previously described [14]. Although FhNEJ typically traverse the intestinal wall within a few hours [19], we extended the stimulation period to up to 24 h, as previously described [28], to account for potential delays in cellular responses owing to the artificial and reductionist nature of the co-culture system.

First, our enzymatic assays revealed that cell culture supernatants from mPSIEC stimulated with both FhNEJ and PLG exhibited increased plasmin activity compared with those from control or single-treated cells, indicating that FhNEJ stimulate plasmin generation in the pericellular space of the mouse small intestine in the presence of host PLG. Since FhNEJ cannot directly cleave and activate PLG [14], and mPSIEC were stimulated in the absence of exogenous addition of either t-PA or u-PA, these results suggest that mPSIEC express the PLG activator(s) required to convert PLG into plasmin, which might have important physiological and pathological implications. Notably, FhNEJ-induced plasmin generation was completely abrogated in the presence of ε-ACA, indicating that this phenomenon depends on the interaction between lysine residues present in PLG-interacting proteins expressed by FhNEJ and the PLG kringle domains, replicating the mechanism of PLG activation utilised by physiologic PLG-Rs.

Since u-PA is expressed by epithelial cells of the small intestine, presumably to loosen intercellular junctions and facilitate epithelial cell turnover at the top of the villi [42], we studied whether the combination of FhNEJ and PLG would increase the availability of this PLG activator in the mPSIEC extracellular space. Results revealed that cell culture supernatants of mPSIEC stimulated with both FhNEJ and PLG contained significantly more u-PA than untreated or single-treated cells, suggesting that plasmin generation in the pericellular space may initiate a positive feedback loop that enhances u-PA release by double-treated cells. These findings indicate that plasmin may enhance fibrinolysis within the small intestinal microenvironment through two complementary mechanisms: the well-established ability of this protease to cleave and activate the zymogen form of u-PA [43, 44] and the stimulation of u-PA secretion by intestinal cells, as demonstrated in the present study. This regulatory mechanism by plasmin is not unprecedented, as plasmin was shown to enhance MMP-12 activity in macrophages by a dual mechanism involving both the activation of this enzyme and the stimulation of its secretion to the extracellular space without a concomitant increase in MMP-12 expression levels, an effect that was demonstrated to depend on plasmin catalytic activity [45].

Plasmin regulates cell migration by degrading ECM components such as collagen type IV and laminin [11, 46], two major structural proteins of the intestinal basement membrane [25]. Therefore, we assessed whether FhNEJ-induced plasmin generation promotes intestinal ECM degradation by measuring total collagen levels in cell culture supernatants of mPSIEC treated with FhNEJ, PLG, or both. Decreased collagen levels in cell culture supernatants were interpreted as increased degradation of this ECM protein, as previously described [47]. Our results revealed that collagen levels slightly decreased in mPSIEC supernatants treated with either FhNEJ or PLG, but the reduction became significant only when both were combined, indicating that FhNEJ-induced plasmin generation promotes collagen degradation in the small intestinal microenvironment. This notion is further supported by the observation that plasmin efficiently degraded the ECM coating solution employed for mPSIEC culture. Although the interaction between helminth parasites and the host fibrinolytic system is well-established and has long been proposed to facilitate tissue migration through enhanced ECM degradation [1–3], to our knowledge, this is the first experimental demonstration of increased ECM degradation resulting from parasite-induced plasmin generation in a trematode, as this phenomenon had only been shown in the nematode *Dirofilaria immitis* [47]. Considering the potent collagenolytic properties of FhCL3 [48, 49], which is highly expressed and secreted by FhNEJ [50], the residual, non-statistically significant collagen degradation that we detected in cell culture supernatants from mPSIEC only treated with FhNEJ is somewhat unexpected. However, this could be explained by the fact that the commercial kit that we used in our experiments fails to detect the specific collagen isoforms that are degraded by FhCL3, that such isoforms are not present in the ECM coating employed here to culture mPSIEC, or that the extent of collagen degradation by FhCL3 under physiologic conditions is less pronounced than that observed in in vitro experiments performed in the absence of host cells [48, 49].

In addition to directly degrading ECM components, plasmin activates the precursor zymogens of MMP-1, MMP-2, MMP-3, MMP-9, MMP-10, MMP-12, and MMP-13, either via direct cleavage or by activating other MMPs that, in turn, cleave and activate the MMP precursor [51–58]. MMPs are highly proficient at degrading ECM components [12], and given their close relationship with the fibrinolytic system [5], we addressed whether FhNEJ-mediated plasmin generation would stimulate MMP release by mPSIEC [45], which could facilitate FhNEJ trans-intestinal migration by expanding the arsenal of enzymes that are available in the extracellular space for ECM degradation. Our results showed that FhNEJ substantially stimulate the secretion of MMP-3, MMP-8, MMP-12, and pro-MMP-9 by mPSIEC, a response that was not further stimulated in the presence of PLG, indicating that FhNEJ-induced plasmin generation does not affect the secretion of these enzymes by host cells. Since MMP-like enzymes in the FhNEJ secretome constitute only 0.04% of the total protein content [59], we believe that their contribution to the measured MMP levels in cell culture supernatants is likely negligible. It remains to be addressed whether FhNEJ-induced plasmin generation in the intestinal epithelium activates MMP precursors that could contribute to the observed collagen degradation, potentially facilitating the migration of these parasites across the host intestine.

Since plasmin influences biological processes not only by regulating the activity of proteolytic enzymes but also by modulating intracellular signalling pathways that culminate in changes in gene expression [60–63], we conducted a proteomic analysis in whole-cell lysates of mPSIEC treated with both FhNEJ and PLG to investigate how these cells respond to elevated plasmin levels in the pericellular space. To this end, we applied a stringent statistical pipeline [33] combined with an analysis of interaction effects to identify proteins with statistically different abundances in FhNEJ + PLG-treated mPSIEC compared with untreated cells that are specific to the combination of FhNEJ and PLG. This analysis included elastic-net penalised regression, which has emerged as a powerful and validated approach for robust feature selection, enabling the reliable identification of condition-specific gene and protein expression signatures in diverse biomedical fields [64–67]. In addition, differential expression was performed with the limma package, which applies a combination of statistical principles that outperform ordinary *t*-tests in proteomics data analysis when sample sizes are small [36, 68]. In parallel to this, we also compared the proteomes of FhNEJ-treated and FhNEJ + PLG-treated mPSIEC to reduce the overall variability and enhance the detection of subtle but biologically meaningful proteomic changes specifically induced by the combined action of FhNEJ and PLG that may be overlooked in the broader untreated versus FhNEJ + PLG-treated contrast. Supporting the accuracy of this approach, only the comparison between FhNEJ-treated and FhNEJ + PLG-treated cells captured PLG, which is expected to be present at residual and almost undetectable levels, as significantly over represented in whole-cell lysates of double-treated mPSIEC (Table 2).

Some of the DEPs resulting from the interaction between FhNEJ and PLG were involved in biological processes that could be beneficial to FhNEJ trans-intestinal migration and survival within the mammalian organism, including cell adhesion and migration, cytoskeleton dynamics, ECM remodelling, mucosal immunity and fibrinolysis. For instance, we identified Cap1, serpin b5, Rab3b, and Actn4 as differentially regulated upon mPSIEC stimulation with both FhNEJ and PLG. Cap1 is involved in cell adhesion and motility through the regulation of actin cytoskeleton dynamics [69], serpin b5 is a non-inhibitory serpin that regulates u-PA/u-PAR-mediated cell adhesion [70] and Rab3b is required for the distribution of the major tight junction (TJ) adaptor protein zonula occludens-1 to the apical membrane of epithelial cells [71, 72]. Similarly, depletion of Actn4 inhibits the recruitment of occludin to TJs, thereby disrupting the formation of functional intercellular junctions [73]. Differential expression of these proteins in mPSIEC stimulated with both FhNEJ and PLG indicates that plasmin might regulate parasite adhesion to the intestinal epithelium and modulate epithelial junction dynamics, potentially compromising epithelial barrier integrity and facilitating FhNEJ migration across the host intestine.

In addition, some of the DEPs identified in mPSIEC incubated with both FhNEJ and PLG play important roles in ECM remodelling. Among others, we found differential expression of Mmp-11 and Timp3 in double-treated cells, suggesting that FhNEJ-induced plasmin generation alters the protease/anti-protease balance of the MMP system to ultimately regulate ECM degradation. Overexpression of Mmp-11 is particularly compelling, as this metalloproteinase degrades collagen type IV [74], a major structural component of the intestinal basement membrane [25]. In the context of ECM remodelling, it is noteworthy that we also detected downregulation of the ECM protein fibronectin (Fn1) in whole-cell lysates of mPSIEC incubated with both FhNEJ and PLG. Decreased Fn1 expression in double-treated mPSIEC aligns with Mmp-11 overexpression in these cells, as MMP-11 was shown to suppress fibronectin gene expression in an in vivo zebrafish model of ECM remodelling [75].

Another notable functional aspect of the interaction between FhNEJ and PLG, highlighted by our proteomic analysis, is the identification of proteins linked to host immune responses and, potentially, to FhNEJ immune evasion mechanisms. Among others, we found downregulation of Sema3d, Pigr, and the complement molecule C7 in mPSIEC treated with both FhNEJ and PLG. Sema3d has been recently shown to promote pancreatic cancer progression by stimulating macrophage polarisation into a protumourigenic M2 phenotype [76]. Since M2 polarisation is a hallmark of anti-*Fasciola* immune responses [77], this finding offers novel insights into how these responses might be regulated at the intestinal level during infection with FhNEJ. In the context of FhNEJ trans-intestinal migration, the downregulation of Pigr in mPSIEC upon stimulation with FhNEJ and PLG is particularly noteworthy, as this protein is a key regulator of mucosal immunity by facilitating the translocation of immunoglobulin A (IgA) to the apical compartment of the intestinal epithelium [78]. In fact, mice lacking Pigr expression show increased bacterial infiltration into the liver due to impaired IgA-mediated antimicrobial defence in the gut [79].

The complement molecule C7 is essential for the assembly of the lethal, pore-forming membrane attack complex, a key effector in antibacterial defence. Previous studies had shown that *F. hepatica* expresses molecules that inhibit complement proteases, thereby evading complement-mediated responses [80]. To the best of our knowledge, the present study provides the first evidence that FhNEJ modulate the expression of C7 in mouse intestinal epithelial cells in a PLG-dependent manner. Intriguingly, the complement protein C3 was also downregulated in FhNEJ-treated mPSIEC compared with untreated controls (Supplementary Table S1), suggesting that FhNEJ modulate the expression of complement molecules through plasmin(ogen)-dependent and independent mechanisms. The S1pr2 is another immune-related protein that we identified as differentially regulated in double-treated mPSIEC. This protein regulates the onset of type 2 immune responses in the lung by inhibiting the secretion of interleukin 33 (IL-33) by lung macrophages [81]. IL-33 is also secreted by intestinal epithelial cells and has recently been shown to represent an indispensable trigger of intestinal type 2 immune responses that lead to successful gastrointestinal nematode expulsion [82]. Based on this, the observed S1pr2 overexpression in mPSIEC upon stimulation with both FhNEJ and PLG may serve to reduce IL-33 release by intestinal epithelial cells and suppress the onset of anti-helminth immunity in the intestine, providing an intriguing direction for future investigation.

Moreover, our proteomic analysis indicated that plasmin itself might also be regulating fibrinolysis in the small intestine tissue microenvironment, as double-treated mPSIEC differentially regulated the expression of two major fibrinolysis inhibitors: A2m and TAFI, which were downregulated and overexpressed, respectively, in mPSIEC upon stimulation with FhNEJ and PLG. This finding suggests that fine-tuning fibrinolysis may benefit the parasite by balancing proteolytic activity such that it facilitates tissue penetration while preventing excessive ECM degradation, which could inadvertently lead to severe tissue damage and exacerbated inflammatory responses in the intestine.

Finally, we employed a mouse model of acute fasciolosis [29] to conclusively determine whether the fibrinolytic system is involved in the intra-mammalian migration of *F. hepatica* juveniles during early infection. To that end, mice were injected with either PBS or PAI-1 to inhibit the fibrinolytic route prior to infection with *F. hepatica* metacercariae. We utilised a recombinant and stable-mutant version of PAI-1, as previously described [40], because the wild-type inhibitor has a very short half-life of approximately 2 h [83]. A time point of 8 days post-infection was selected for quantifying infection success on the basis of previous studies indicating that juvenile recovery from the livers of infected mice is highest at this time [29]. Results showed that the number of liver-migrating flukes in PAI-1-treated mice was significantly lower than that observed in control mice but only in the experiment conducted on day 1.

Since differences in juvenile counts across experimental days were observed only in PAI-1-treated mice, but not in their control counterparts, we suspected that variations in PAI-1 treatment efficacy between days might explain these results. To investigate this possibility, fibrinolysis was evaluated by measuring the levels of active PAI-1 and t-PA in peritoneal fluids collected immediately after euthanasia. Although the differences in active PAI-1 and t-PA levels between PAI-1-treated and control mice on day 1 were more consistent with the expected inhibition of fibrinolysis than those observed in mice from day 2, these differences were small and did not reach statistical significance. This is likely due to the fact that peritoneal fluids were collected 8 days after PAI-1 treatment, exceeding the maximum half-life of 6 days achieved for this factor through random mutagenesis [83]. Altogether, these results highlight the complexity of working with genetically diverse organisms such as *F. hepatica*, which likely constitutes one of several factors contributing to the inconsistent and variable outcomes reported in vaccine trials over the past decades [84, 85]. This variability not only hinders data interpretation but also raises ethical concerns regarding the use of large animal cohorts to overcome inter-individual heterogeneity and meet significance thresholds that are inherently arbitrary. Notwithstanding these points, and despite the observed inter-experimental variability, we believe that these results provide the first functional preliminary evidence that host fibrinolysis supports juvenile migration in vivo, offering a solid foundation for future research in this area.

## Conclusions

This study demonstrates that plasmin is generated in the pericellular space of mouse intestinal epithelial cells in the presence of FhNEJ and host-derived PLG. This process correlates with increased collagen degradation and u-PA release into the extracellular environment, potentially facilitating FhNEJ migration across the intestinal wall. In addition, FhNEJ-induced plasmin generation triggers changes in the expression levels or abundance of proteins involved in biological processes that could be critical during the earliest stages of infection, including cell adhesion and migration, ECM remodelling, immune evasion, and fibrinolysis. Finally, despite the inherent challenges associated with in vivo modelling of *F. hepatica* infections, our findings provide the first functional preliminary evidence supporting the long-proposed role of the fibrinolytic system in facilitating the migration of *F. hepatica* juveniles through mammalian tissues. Collectively, these results underscore the contribution of the fibrinolytic system in parasite dissemination during early stage fasciolosis, providing interesting insights into host–parasite relationships established in the initial stages of infection and highlighting potential avenues for future research in this area.

## Supplementary Information

Additional file 1.

## Data Availability

Data are provided within the manuscript or supplementary information files.

## Abbreviations

Actn4: Actinin-4
ANOVA: Analysis of variance
A2m: Alpha-2 macroglobulin
Cbp2: Carboxipeptidase
C7: Complement molecule 7
DEP: Differentially expressed protein
ECM: Extracellular matrix
ELISA: Enzyme-linked immunosorbent assay
FC: Fold change
FDR: False discovery rate
FhNEJ: *F. hepatica* newly excysted juvenile
Fn1: Fibronectin 1
IgA: Immunoglobulin A
IL-33: Interleukin 33
IQR: Interquartile range
MMPs: Matrix metalloproteinases
MMP11: Matrix metalloproteinase 11
mPSIEC: Mouse primary small intestinal epithelial cells
MS/MS: Tandem mass spectrometry
PBS: Phosphate-buffered saline
Pigr: Polymeric immunoglobulin receptor
PLG: Plasminogen
PLG-R: Plasminogen receptor
PLS-DA: Partial least squares discriminant analysis
pro-u-PA: Precursor of the urokinase-type plasminogen activator
Sema3d: Semaphorin 3D
SDS: Sodium dodecyl sulphate
SDS-PAGE: Sodium dodecyl sulphate–polyacrylamide gel electrophoresis
SD: Standard deviation
SEM: Standard error of the mean
S1pr2: Sphingosine 1-phosphate receptor 2
u-PA: Urokinase-type plasminogen activator
TAFI: Thrombin-activatable fibrinolysis inhibitor
Timp3: Tissue inhibitor of metalloproteinase 3
TJ: Tight junction
ε-ACA: 6-Aminocaproic acid
