# Supplementary material for: Early host–parasite interaction models reveal a key role for fibrinolysis in Fasciola hepatica intestinal migration
Source: Parasit Vectors. 2025 Sep 24;18:387. doi: 10.1186/s13071-025-06992-9 (PMC12462368; doi:10.1186/s13071-025-06992-9)
Supplement: Supplementary file 1 — Additional file 1. [file 13071_2025_6992_MOESM1_ESM.pdf]

Supplementary information

Additional file 1: Fig. S1. Feature selection by elastic-net penalised regression improves sample clustering. An elastic-net penalised regression model was performed to select for proteins that are specifically expressed in each experimental condition. The figures show heatmap representations of protein expression levels (normalised to Z-score) of all the proteins identified (left panels) or proteins selected as specific to each experimental condition after feature selection through elastic-net regularised regression (right panels), for selected, biologically-relevant pairwise comparisons: untreated mPSIEC vs. mPSIEC treated with FhNEJ (**A**); untreated mPSIEC vs. mPSIEC treated with both FhNEJ and PLG (**B**); and FhNEJ-treated mPSIEC vs. mPSIEC treated with both FhNEJ and PLG (**C**). Heatmaps created with R software.

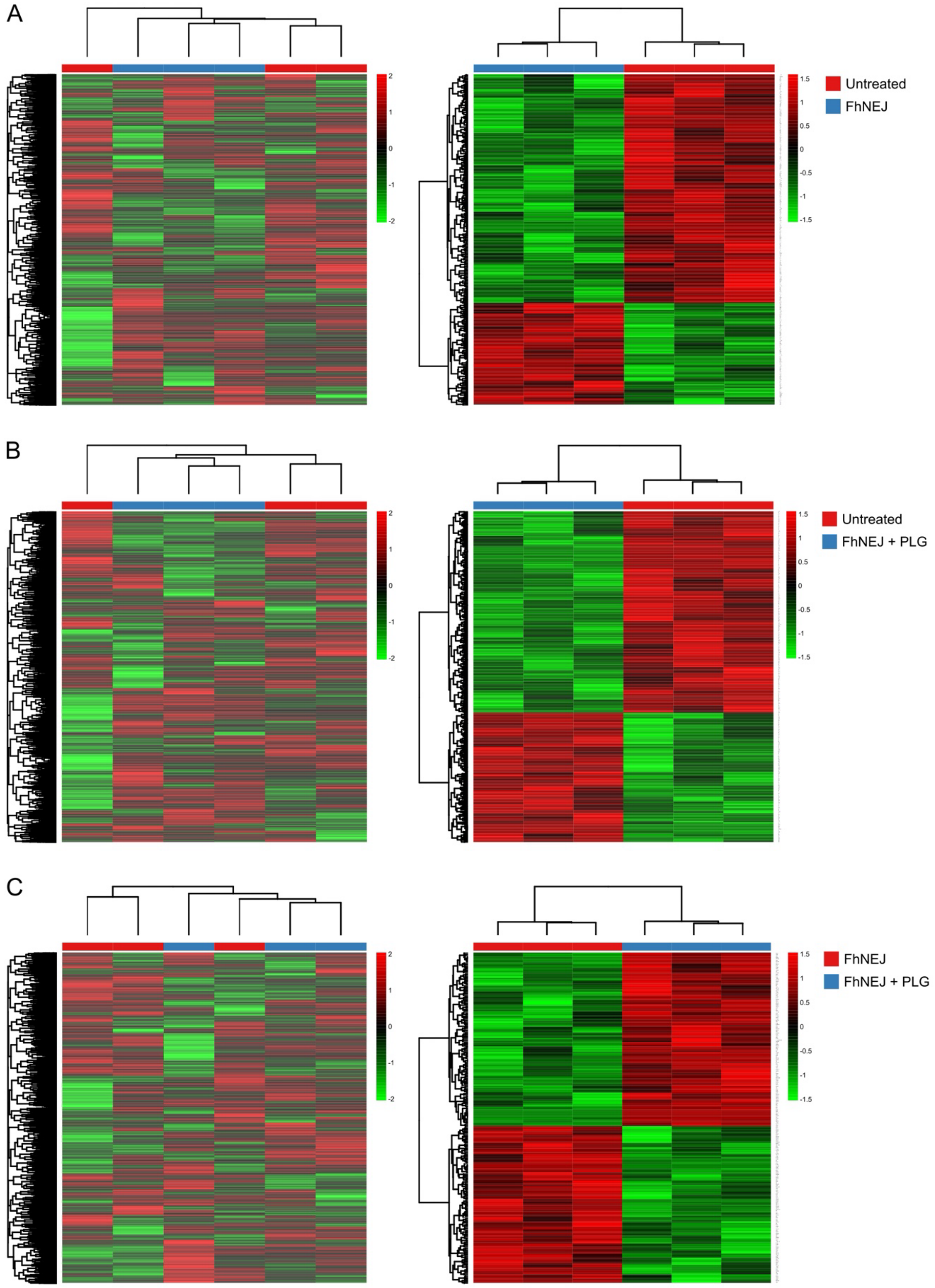

A
Untreated
FhNEJ
B
Untreated
FhNEJ + PLG
C
FhNEJ
FhNEJ + PLG

Additional file 2: Fig. S2. PLS-DA analysis of selected pairwise comparisons. PLS-DA was performed to identify the proteins most relevant for distinguishing between experimental conditions. Only those elastic-net pre-selected proteins that contributed substantially to the newly projected bidimensional space (as determined by vip>1.5) were considered as specific to each experimental condition. Plots are shown for selected, biologically-relevant pairwise comparisons: untreated mPSIEC vs. FhNEJ-treated mPSIEC (top left); untreated mPSIEC vs. mPSIEC treated with both FhNEJ and PLG (top right); and FhNEJ-treated mPSIEC vs. mPSIEC treated with both FhNEJ and PLG (bottom). Plots created with R software.

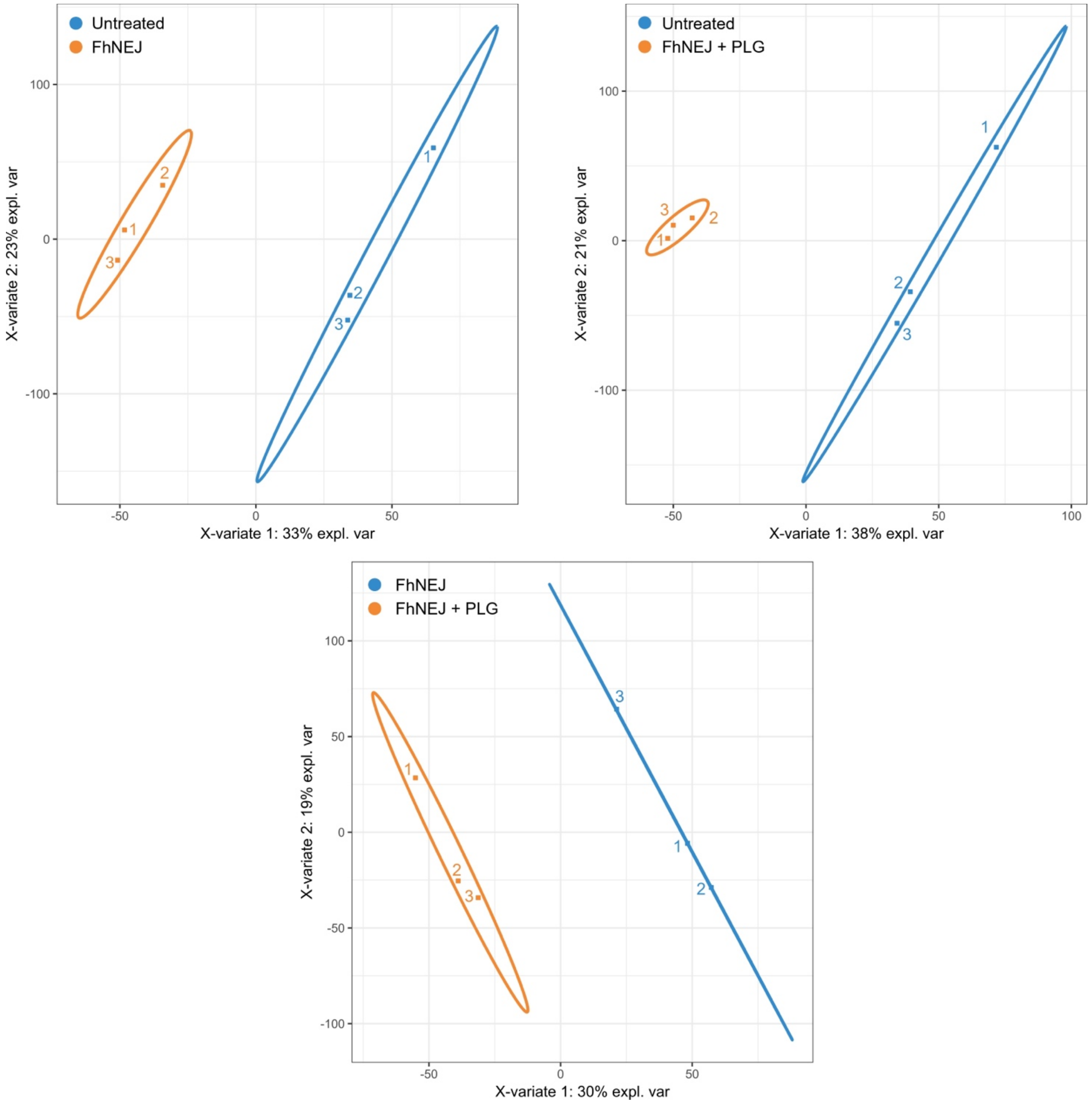

Additional file 3: Fig. S3. (**A**) The effect of plasmin on the detection of total collagen was determined using the same commercial kit as in Figure 2B by pre-incubating total collagen with 2 µM plasmin for 24 hours at 37 °C followed by enzymatic digestion and detection of N-Gly terminal peptides, as specified by the manufacturer's instructions. Total collagen left undigested ("Undigested") or digested without plasmin pre-incubation ("+ Enzyme") served as controls. Bars indicate the mean of three technical replicates ± SD (***p≤0.001, ****p≤0.0001; one-way ANOVA followed by Tukey post hoc analysis of pairwise comparisons). RFU, relative fluorescent units (excitation 380 nm, emission 460 nm). (**B**) Zymography showing plasmin-mediated degradation of the ECM coating used for mPSIEC culture. Different amounts of plasmin (2 µM, 0.5 µM, and 0.1 µM) were loaded onto 10% SDS-PAGE gels prepared using mPSIEC coating solution instead of water. Light bands (arrowhead) corresponding to degradation of the mPSIEC coating solution by plasmin were revealed by Coomassie brilliant blue staining.

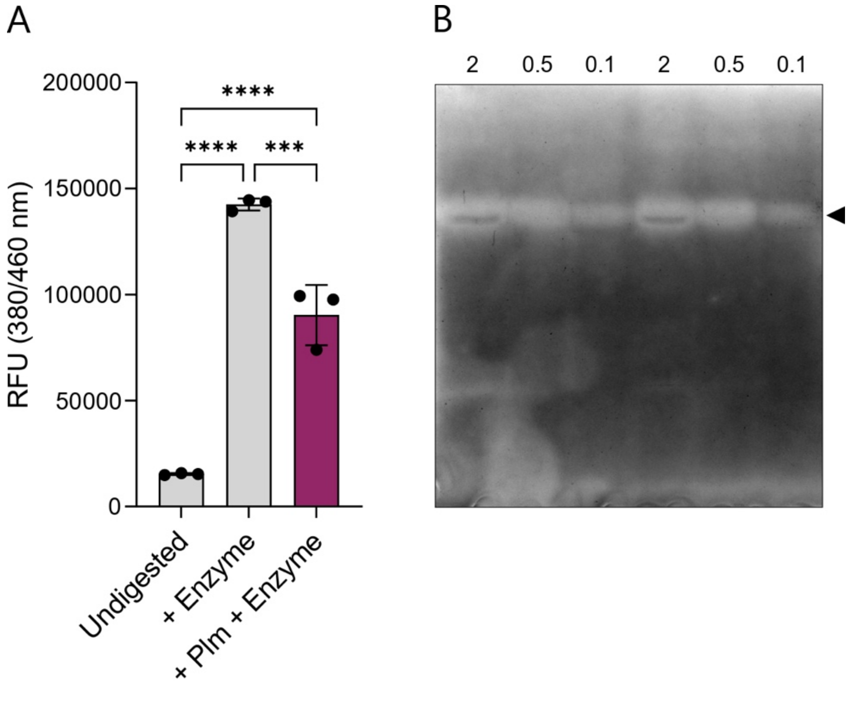

Additional file 4: Fig. S4. Fluke recovery in control and PAI-1-treated mice across experimental days. The number of flukes recovered from control (**A**) and PAI-1-treated (**B**) mice was plotted separately to assess possible variation in intra-group responses across days. Data points represent the number of juveniles recovered in the liver of each mouse, and bars indicate the mean ± SEM.

Asterisks indicate significant differences between experimental conditions (ns, not significant; **p≤0.1; Mann-Whitney U test).

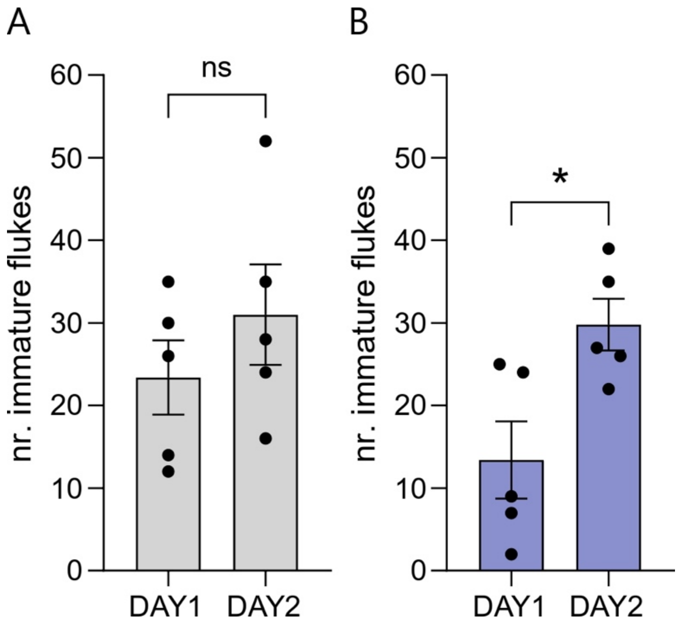

Additional file 5: Fig. S5. Analysis of PAI-1 and t-PA activities in peritoneal fluids of *F. hepatica*-infected mice. Eight days after challenge with *F. hepatica* metacercariae, mice were euthanised and the levels of active PAI-1 (**A**) and t-PA (**B**) in peritoneal fluids were quantified by ELISA using commercially-available kits. Data points represent the levels of active PAI-1 (**A**) or t-PA (**B**) in the peritoneal fluid of each mouse, and bars indicate the mean ± SEM. None of the differences were significant (Mann-Whitney U test).

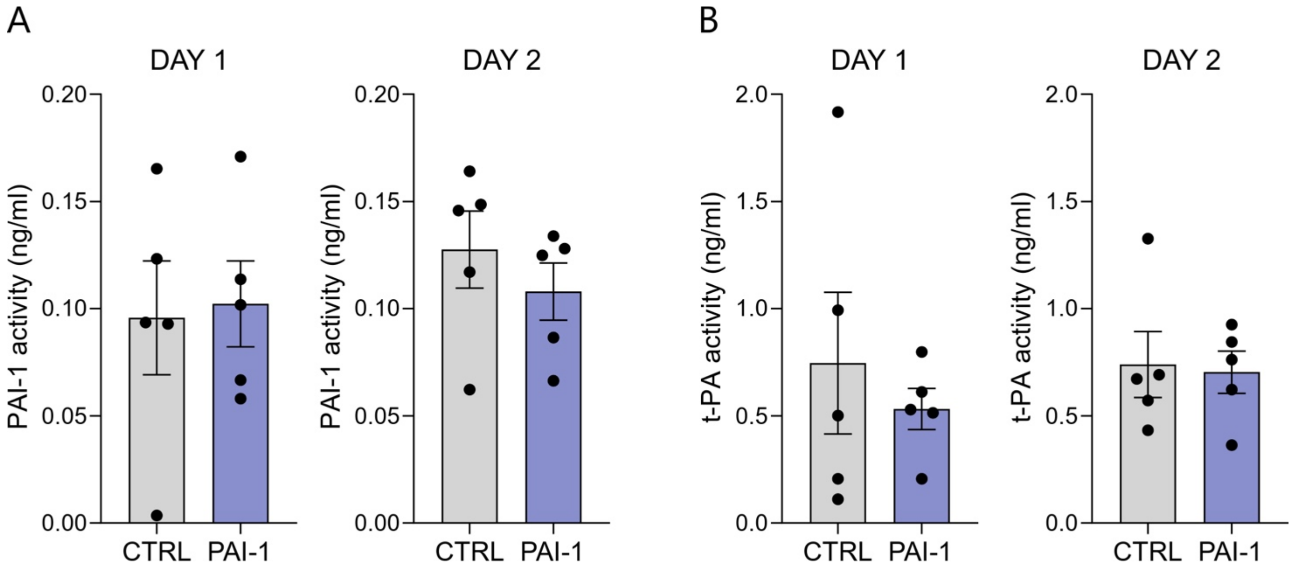

Additional file 6: Table S1. DEPs in untreated vs. FhNEJ-treated mPSIEC. A negative fold-change indicates that proteins were overexpressed in FhNEJ-treated vs. untreated mPSIEC. The opposite is true when the fold-change is positive.

Protein	logFC	adjPVal	Protein	logFC	adjPVal
Rpl32	-11.656	1.73E-08	Itih2	1.563109	0.023871
Cdc42	-11.6507	2.79E-08	C3	1.606383	0.038488
Abpa12a	-8.81047	3.38E-05	Cpn1	7.886917	3.38E-05
S1pr2	-8.67745	2.79E-08	Map6	8.331462	4.68E-06
Plin2	-1.07837	0.000559	Qsox1	8.616831	1.45E-06
Jagn1	-0.46458	0.049104	Itih3	8.676878	1.73E-08
Ttyh3	0.308236	0.049048	Hp	9.253502	6.43E-05
Cdca8	0.424581	0.032809	Hexim2	10.7249	7.17E-07
Pzp	0.891437	0.008727	Alb	13.12146	0.000149
Bcl7b	1.024835	0.04689			

Additional file 7: Table S2. Two-way ANOVA of the number of recovered *F. hepatica* juvenile flukes in the livers of control and PAI-1-treated mice eight days after challenge with *F. hepatica* metacercariae. SS, sum of squares; DF, degrees of freedom; MS, mean square; F, F statistic; DFn, degrees of freedom of the numerator; DFd, degrees of freedom of the denominator.

Two-way ANOVA	Ordinary				
Alpha	0.05				
Source of Variation	% of total variation	P value	P value summary	Significant?	
Interaction	3.522	0.3642	ns	No	
Day	26.19	0.0215	*	Yes	
Treatment	5.704	0.2519	ns	No	
ANOVA table	SS	DF	MS	F (DFn, DFd)	P value
Interaction	96.8	1	96.8	F (1, 16) = 0.8725	P=0.3642
Day	720	1	720	F (1, 16) = 6.489	P=0.0215
Treatment	156.8	1	156.8	F (1, 16) = 1.413	P=0.2519
Residual	1775	16	111		
